# Efficacy of Cross-Linked Collagen Membranes for Bone Regeneration: In Vitro and Clinical Studies

**DOI:** 10.3390/bioengineering12080876

**Published:** 2025-08-14

**Authors:** Se-Hoon Baek, Byoung-Eun Yang, Sang-Yoon Park, Sung-Woon On, Kang-Min Ahn, Soo-Hwan Byun

**Affiliations:** 1 Department of Oral and Maxillofacial Surgery, Hallym University Sacred Heart Hospital, Anyang 14066, Republic of Korea; 2078oms@gmail.com (S.-H.B.); face@hallym.ac.kr (B.-E.Y.); psypjy0112@naver.com (S.-Y.P.); 2 Graduate School of Clinical Dentistry, Hallym University, Chuncheon 24252, Republic of Korea; drummer0908@hallym.or.kr; 3 Institute of Clinical Dentistry, Hallym University, Chuncheon 24252, Republic of Korea; 4 Dental AI-Robotics Center, Hallym University Sacred Heart Hospital, Anyang 14066, Republic of Korea; 5 Department of Oral and Maxillofacial Surgery, Hallym University Dongtan Sacred Heart Hospital, Hwaseong 18450, Republic of Korea; 6 Department of Oral and Maxillofacial Surgery, Seoul Asan Medical Center, Seoul 05505, Republic of Korea; ahnkangmin@gmail.com

**Keywords:** cross-linking methods, collagen membrane, guided bone regeneration (GBR), cone-beam computed tomography (CBCT)

## Abstract

This study aimed to evaluate the efficacy of cross-linked collagen membranes. Two types of collagen membranes were compared: a non-cross-linked collagen membrane (group A) and a cross-linked (group B) collagen membrane. In the in vitro study, the degradation rate in the presence of collagenase, the tear strength of the membranes, and the cytotoxicity of the cross-linked collagen membrane were evaluated. A total of 57 participants with cystic defects were randomized to undergo guided bone regeneration (GBR) using either membrane. Graft volume and new bone formation were measured by cone-beam computed tomography after 6 months of follow-up. In vitro findings revealed that the cross-linked collagen membrane retained more than 20% of its relative weight after 12 h. Meanwhile, the non-cross-linked collagen membrane exhibited complete degradation after 6 h. Clinically, no significant differences were observed between the groups in terms of graft resorption, new bone formation, and overall bone regeneration. These results indicate that cross-linking has comparable biocompatibility and enhances physical properties, including tear strength and resistance to degradation. However, clinical outcomes related to bone regeneration were not significantly different between cross-linked and non-cross-linked collagen membranes. Further research is warranted to determine the benefits of cross-linked collagen membranes in GBR procedures.

## 1. Introduction

Bone defects in the oral and maxillofacial region may result from various causes, including benign or malignant tumors, jaw cysts, tooth extractions, congenital deformities such as cleft lip and palate, and traumatic injuries [1,2,3,4,5]. Such defects are associated not only with esthetic concerns but also with functional impairments, including difficulties in mastication and speech [6,7]. Therefore, effective reconstruction of these defects is essential for restoring both appearance and function [8,9]. Guided bone regeneration (GBR) is one of the most widely adopted approaches for restoring jawbone defects, particularly through the application of particulate bone grafts in combination with barrier membranes. Favorable outcomes have been reported with this approach, especially in the augmentation of bone height and width [10,11].

In GBR procedures, either resorbable or non-resorbable membranes are employed to establish a barrier between the bone defect and the periosteum. This barrier maintains the space of the defect and prevents the ingrowth of rapidly proliferating soft tissue cells; as a result, osteoprogenitor cells can infiltrate the defect site and promote proper osteogenesis [12]. The choice between resorbable and non-resorbable membranes is determined based on the required duration of barrier function. The ideal membrane for GBR should possess biocompatibility, effective cell occlusion, integration with host tissues, clinical manageability, space-maintaining capacity, and sufficient mechanical and physical strength [13,14].

The most commonly used absorbable membranes are collagen membranes. Collagen, a major component of the extracellular matrix, has been widely utilized in scaffold fabrication and has demonstrated significant potential in tissue regeneration [15]. These membranes are favored in clinical practice, as they are readily absorbed by the body without complications and allow for easy handling. Type I collagen, the predominant component of periodontal connective tissue, has been adopted as the principal material in several commercially available collagen membranes. Collagen-based materials provide multiple advantages, such as hemostasis, chemotaxis for the periodontal ligament and gingival fibroblasts, low immunogenicity, ease of manipulation, and the ability to augment tissue thickness [16]. Therefore, absorbable membranes are frequently preferred by clinicians. However, the rapid degradation of collagen within the body may compromise its ability to protect against bone defects and support bone formation. The loss of structural integrity due to rapid biodegradation remains a major limitation of collagen membranes.

Several cross-linking methods have been used to reduce the degradation rate of collagen, thereby prolonging its barrier function [17]. These techniques are generally classified into physical, chemical, enzymatic, and UV-based approaches [18]. Among them, chemical cross-linking methods commonly employ agents such as glutaraldehyde, carbodiimide, and genipin [19]. In many studies, various substances have been used to induce cross-linking within collagen membranes, including agents with considerable cytotoxic potential, such as glutaraldehyde or formaldehyde [20]. Certain studies have indicated that glutaraldehyde-based cross-linking may reduce biocompatibility [21,22]. Residual glutaraldehyde can bind to collagen components after cross-linking, thereby altering cell adhesion [23].

Cross-linking not only slows the degradation of the collagen membranes but also improves their physical properties, such as tear strength, making them more suitable for clinical applications [24]. Opinions on collagen membrane cross-linking vary among researchers [25]. Concerns have been raised regarding the potential toxicity of residual chemical agents used during the collagen cross-linking process. Additionally, some clinicians avoid the use of cross-linked collagen membranes due to changes in their physical properties.

Nevertheless, only a few studies have evaluated the clinical efficacy and safety of cross-linked collagen membranes, leaving clinicians uncertain when selecting them for use. Recent advancements in cross-linking and collagen processing technologies have resulted in the commercialization of various cross-linked collagen membranes. These membranes offer enhanced physical properties and improved clinical usability, highlighting the growing importance of research in this field.

Collagen membranes are currently the most extensively studied and clinically utilized barrier membranes in guided bone regeneration [10]. Accordingly, advancing our understanding of their physical characteristics is essential to optimizing their performance in clinical applications. Alongside these advances, recent studies have also explored synthetic and hydrogel-based membranes, aiming to enhance handling and biological performance. This reflects the growing importance of developing materials with improved physical properties for case-specific clinical application [26].

This study aimed to evaluate whether the cross-linking of collagen membranes enhances their physical properties while preserving biocompatibility. In addition, we aimed to assess the efficacy of cross-linked collagen membranes for GBR through in vitro and clinical studies. To this end, this study performed a degradation test using collagenase and tear strength test with a mechanical testing system, respectively, and conducted a randomized clinical study comparing volumetric bone regeneration between cross-linked and non-cross-linked membranes over a 6-month follow-up period using CBCT imaging.

This manuscript begins with a detailed description of the materials and methodologies employed in both the in vitro evaluations—degradation test, tear resistance test, and cytotoxicity test—and the clinical study protocol. The subsequent section presents the findings from the in vitro experiments alongside the clinical outcomes. These results are then interpreted and contextualized in a comprehensive discussion, culminating in a concise conclusion that highlights the key implications of the study.

## 2. Materials and Methods

### 2.1. In Vitro Study

#### 2.1.1. Degradation Test

A degradation test was performed, following ISO 10993-13 standards. The extent of membrane degradation using collagenase was evaluated for both groups A and B. These membranes were cut into 10 mm × 20 mm pieces for testing, and testing was conducted once for each membrane type (*n* = 1). In an in vitro setting, collagenase type I at a concentration of 50 U/mL was applied to two types of membranes separately, each exposed for 12 h. The preparation method for the collagen solution was as follows: a 1 M Tris-HCl buffer (Elpis biotech, Daejeon, Republic of Korea) was diluted to 0.1 M and supplemented with 5 mM CaCl2 (SAMCHUN, Seoul, Republic of Korea) to prepare the Tris–HCl buffer solution. Collagenase (Sigma Aldrich, MO, USA/Collagenase from Clostridium histolyticum for general use, Type I ≥ 125 CDU/mg solid) was then added to the solution to a final concentration of 50 U/mL. The relative weight of the collagen membranes was then compared over time.

#### 2.1.2. Tear Test

All procedures in this experiment were conducted in accordance with the standardized protocol ASTM D882-09 (tensile rate: 70 mm/min). Both types of collagen membranes were cut into 10 × 30 mm strips, with defect-free specimens selected for testing. An artificial bone block was secured to the upper jig of a Universal Testing Machine (UTM, H50KT, Tinius Olsen, PA, USA) equipped with a 100 N load cell, and the membrane was fastened using a pin. The membrane was positioned at a grip distance of 10 mm, and the tear strength was measured by pulling the specimen at a speed of 70 mm/min until rupture occurred. This procedure was repeated three times for each group.

#### 2.1.3. Cytotoxicity Test

Cytotoxicity was evaluated following ISO 10993-5 and ISO 10993-12 standards. Samples were cut to appropriate sizes, sterilized, and incubated in culture medium (DMEM supplemented with 10% FBS and 1% penicillin) at 37 °C for 24 h (1 mL per 6 cm^2^ of sample surface area) to obtain the eluate. High-density polyethylene (HDPE) and latex were used as negative and positive controls, respectively.

Mouse fibroblast cells (L-929, NCTC clone 929) were seeded at 1 × 10^5^ cells/mL in 12-well plates and incubated for 24 h. After replacing the medium with the prepared eluates, cells were cultured for an additional 48 h. Cell morphology was observed microscopically, and viability was assessed using the CCK-8 assay. After a 2 h incubation with CCK-8 reagent, absorbance was measured at 450 nm. Each sample type was tested in six replicates. According to ISO 10993-5, cell viability below 70% of the negative control was considered cytotoxic.

### 2.2. Clinical Study

#### 2.2.1. Participants

A total of 66 patients were recruited from the Hallym University Sacred Heart Hospital (Anyang, Republic of Korea) and Asan Medical Center (Seoul, Republic of Korea) based on a sample size calculation assuming an effect size of 1.0, with α = 0.05 and 80% power. Patients were recruited between 14 October 2022 and 31 December 2024, with none terminating their participation early. Patients aged >20 years and requiring bone grafting due to severe alveolar bone loss following cyst removal in the maxilla or mandible were considered eligible for the study. Participants who met all the inclusion criteria and did not meet any exclusion criteria were randomly assigned to either group A or group B using statistical randomizing software—R version 4.3.2 (R Foundation for Statistical Computing, Vienna, Austria)—to minimize allocation bias. At Hallym University Sacred Heart Hospital (Anyang, Republic of Korea), 31 participants were initially recruited; however, 2 participants with missing data or visit-related issues were excluded, leaving 29 participants included in the final analysis. Of the 35 participants initially recruited at the Asan Medical Center (Seoul, Republic of Korea), 7 with missing data or hospital visit-related issues were excluded; hence, the remaining 28 participants were included in the final analysis. Safety assessments were conducted during each of the 5 scheduled screening or follow-up visits to monitor for potential adverse reactions such as wound dehiscence, inflammation, or delayed healing. No such events were observed in any of the participants throughout the study period. Patients with anticipated incomplete primary closure following GBR were excluded. Patients were not involved in the design, conduct, or reporting of this study. Written informed consent was obtained from all patients. All procedures were approved by the Institutional Review Boards of both institutions (2022-01-016-014 and 2022-1114), and the trial was registered at cris.nih.go.kr (KCT0008998, 30 November 2023).

#### 2.2.2. Inclusion and Exclusion Criteria

Patients were enrolled in this study based on predefined inclusion and exclusion criteria approved by the Institutional Review Board. The inclusion criteria were as follows: adults aged ≥20 years; patients with severe alveolar bone loss resulting from cyst removal or periodontitis, requiring bone grafting; cases in which primary closure was achievable following grafting; and those who provided written informed consent for participation.

Exclusion criteria included pregnancy; presence of uncontrolled systemic diseases such as diabetes or hypertension; use of medications known to affect bone metabolism within the past 6 months (e.g., bisphosphonates, corticosteroids, or denosumab); untreated gingivitis, periodontitis, or dental caries; history of radiotherapy to the surgical site; bleeding disorders requiring anticoagulant therapy; known allergies to bone graft or membrane materials; heavy smoking (more than 20 cigarettes per day); and any other condition deemed inappropriate by the investigator.

#### 2.2.3. Ethics Approval and Consent to Participate

The study was conducted in accordance with the guidelines of the Declaration of Helsinki and approved by the Institutional Review Board of Hallym University Sacred Heart Hospital (2022-01-016; 31 May 2022) and Seoul Asan Medical Center (2022-1114; 17 August 2022). The patients’ personal information was kept confidential throughout the study or publication process.

#### 2.2.4. Study Design

**Figure 1:**
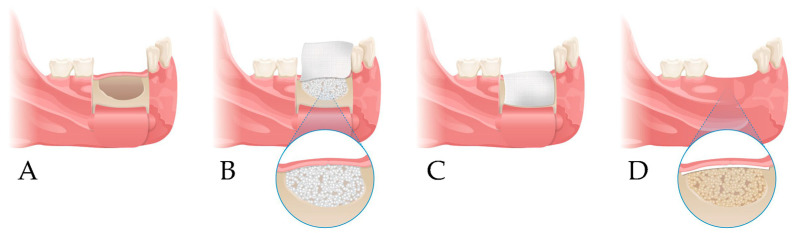
Study design. (**A**) Elevation of a periosteal flap and cyst enucleation; (**B**) xenogenic bone grafting at the bone defect site; (**C**) coverage with collagen membrane; (**D**) follow-up.

**Figure 2:**
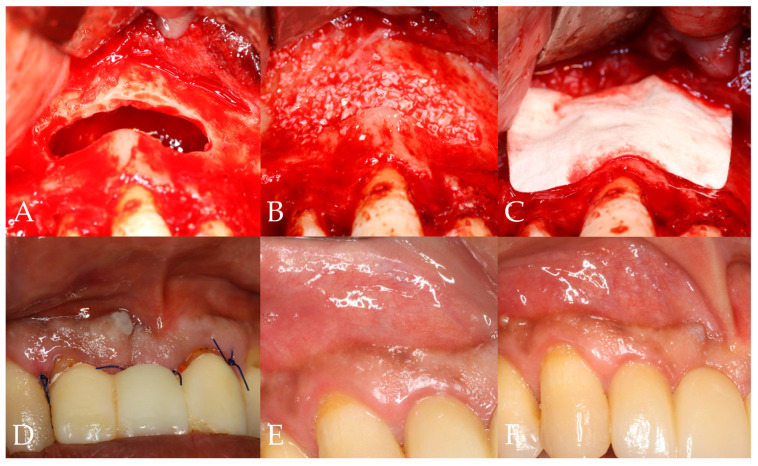
Clinical study protocol. (**A**) Cyst enucleation (2nd visit); (**B**) bone grafting at the defect site (2nd visit); (**C**) coverage with collagen membrane (2nd visit); (**D**) suture removal (3rd visit); (**E**) 3-month follow-up (4th visit); (**F**) 6-month follow-up (5th visit).

This study was designed as a non-inferiority trial to evaluate whether the cross-linked collagen membrane, which was previously questioned for its biocompatibility and bone regenerative potential, could achieve clinical outcomes comparable to those of the non-cross-linked membrane. Participants were randomly allocated into two groups (groups A and B) at a 1:1 ratio during the screening process, with the simple randomization allocating sequence being carried out using statistical software and by an independent investigator. The investigators and surgeons had no access to the random allocation sequence. Following cyst enucleation and bone grafting, the collagen membrane corresponding to each group was cut to an appropriate size and applied over the grafted bone (Figure 1). All surgical procedures were performed according to standard surgical protocols, by experienced board-certified oral and maxillofacial surgeons at each participating hospital. All patients were required to complete a total of five visits, including preoperative screening, surgery, and three postoperative follow-up visits, with suture removal (Figure 2). All participants received standard postoperative care, including antibiotics and analgesics, as part of routine clinical management. Clinical and radiographic evaluations using cone-beam computed tomography (CBCT) were conducted preoperatively, postoperatively, and at the 3-month and 6-month follow-ups [27] (Table 1). Given the relatively short follow-up period and low risk of harm, no interim analyses or stopping rules were planned.

#### 2.2.5. Radiological Evaluation

**Figure 3:**
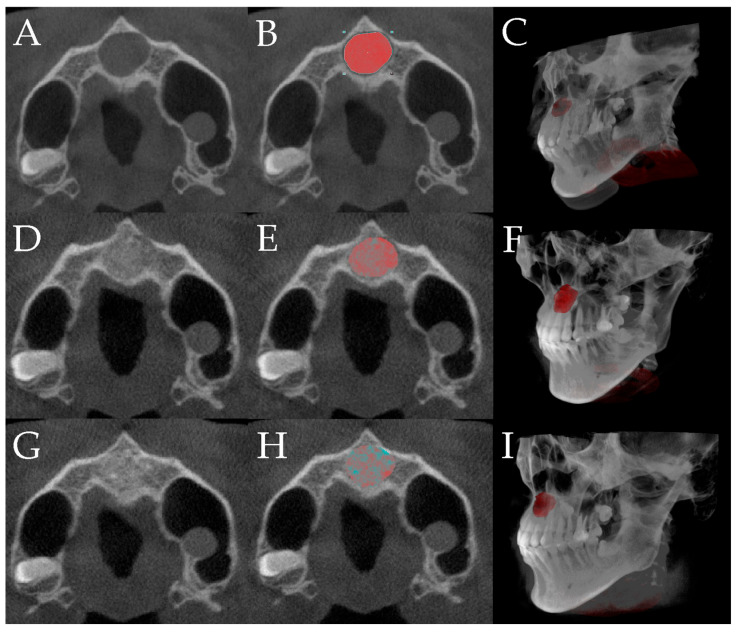
The entire volumetric evaluation process, including segmentation and three-dimensional reconstruction of the defect (red-highlighted), grafted bone, and newly formed bone (grafted bone + newly formed bone; green-highlighted) at three time points (3DSlicer, Slicer Community, TX, USA): preoperative (**A**–**C**), immediate postoperative (**D**–**F**), and 6-month follow-up (**G**–**I**).; (**A**) preoperative image; (**B**) preoperative distinction of defect area; (**C**) preoperative 3D construction of the defect area; (**D**) immediate postoperative image; (**E**) postoperative delineation of the grafted bone area; (**F**) postoperative 3D reconstruction of the grafted bone area; (**G**) image at the 6-month follow-up; (**H**) delineation of the grafted and newly formed bone area at the 6-month follow-up; (**I**) 3D reconstruction of the grafted and newly formed bone area at the 6-month follow-up.

The proportion of bone graft resorption was assessed by calculating the ratio of the bone graft volume immediately after surgery to that at postoperative 6-month follow-up, based on data from 46 patients across both institutions. CBCT images obtained preoperatively, immediately postoperatively, and at the 6-month follow-up were analyzed using the 3DSlicer program (Slicer Community, TX, USA). The area of the grafted bone was outlined on each CBCT layer, and the three-dimensional (3D) volumes were calculated by combining the total marked CBCT layers (Figure 3).

The bone defect volume was segmented from preoperative CBCT images by delineating the radiolucent area within the jaw bone. Because CBCT resolution does not allow for reliable discrimination between newly formed bone and residual graft, the segmented high-density areas were evaluated as a combined volume of mineralized tissue. The grafted bone volume was calculated from the immediate postoperative CBCT by identifying the augmented area. To evaluate newly formed bone, the same region of interest was assessed on the 6-month follow-up CBCT. The reduction in the total defect volume compared to the postoperative scan was interpreted as new bone formation. In addition, high-density areas presumed to represent mineralized tissue (including residual graft and new bone) were segmented using an automatic thresholding algorithm, integrated within the Segment Editor module, and modified with radiographic expert assessment. This approach allowed for objective and reproducible identification of mineralized tissue within the original defect volume (Supplementary Figure S1).

Two parameters were measured to assess bone regeneration at the 6-month follow-up: (1) the volume ratio of new bone formed at the defect site and (2) the proportion of new bone formation relative to the total grafted and regenerated tissue. The volume change ratio was calculated by dividing the 3D volume of the remaining grafted bone and newly formed bone at the 6-month follow-up by the 3D volume of the grafted bone measured on postoperative day 0. The volume ratio of new bone formed in the two groups at the 6-month follow-up was determined by calculating the ratio of the remaining grafts and newly formed bone at the 6-month postoperative follow-up and the defect site volume. The proportion of new bone formation was determined by calculating the ratio of the grafted bone on op day and the combined volume of the remaining grafts and newly formed bone at the 6-month postoperative follow-up.

All CBCT scans were obtained using the same CBCT device (Dentium Rainbow CT; Dentium Co., Suwon, Republic of Korea) under standardized imaging conditions, and all image analyses were performed by a single radiographic evaluator with expertise in CBCT-based assessment; the evaluator was blinded to group allocation throughout the analysis process.

#### 2.2.6. Statistical Analysis

All randomized participants were included in the statistical analysis. Continuous variables were analyzed using independent-sample t-tests, whereas categorical variables were analyzed using the chi-square test. A generalized linear model was used to identify significant differences between the groups. No subgroup or sensitivity analyses were performed. All statistical analyses were performed using IBM SPSS Statistics Version 22.0 (SPSS Inc., IL, USA). All statistical comparisons were conducted at a significance level of 0.05. No important changes were made to the trial methods, prespecified outcomes, or analyses after trial commencement.

## 3. Results

### 3.1. In Vitro Study

#### 3.1.1. Degradation Test

**Figure 4:**
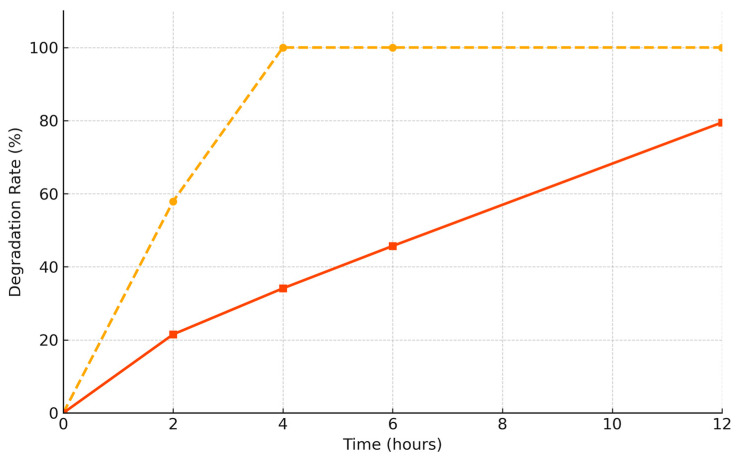
Remaining membrane weight (%) over time; group A (non-cross-linked membrane; yellow dashed line); group B (cross-linked membrane; orange line); group B retained more than 20% of its original mass even at 12 h, whereas group A was fully degraded by 4 h.

During the degradation test, the relative weight of group A decreased to 0% after 4 h. By contrast, group B retained more than 20% of its weight after 12 h (Figure 4) (Table 2). One sample per membrane type was used (*n* = 1) for this test.

#### 3.1.2. Tear Test

The tear strengths in group A and B were 4.0 ± 0.6 N and 18.4 ± 1.1 N, respectively. Thus, the tear strength of the cross-linked collagen membrane was 4.6 times higher than that of the non-cross-linked collagen membrane (Table 2).

#### 3.1.3. Cytotoxicity Test

In the cytotoxicity experiment, the cytotoxicity of group B was measured alone and compared to both positive and negative control groups. The negative control group showed approximately 84.6% cell viability, as well as an absorbance of over 70%, indicating that group B exhibited clinically safe levels of cytotoxicity (Table 3).

### 3.2. Radiological Evaluation

#### 3.2.1. Total Volume Change

In this experiment, the extent of defect was compared between the two groups to assess the differences in the degree of volume change from the immediate postoperative day 0 to the 6-month follow-up. The mean and standard deviation were compared to assess the volume changes observed at the 6-month follow-up relative to the immediate postoperative day 0 measurements. The mean volume changes in group A and B were 84.13 ± 12.42% and 83.91 ± 11.34%, respectively, with no significant difference between the two groups (*p* > 0.05).

#### 3.2.2. Volume Ratios of New Bone Formation

In this experiment, the amount of newly formed bone was compared between the two groups to determine whether differences existed in the extent of new bone formation. The mean and standard deviation were compared to assess the changes observed at 6-month follow-up postoperatively relative to the immediate postoperative day 0 measurements. The mean volume ratios of new bone formation in group A and B were 86.57 ± 11.91% and 85.04 ± 18.80, respectively, with no significant difference between the two groups being observed (*p* > 0.05).

## 4. Discussion

In this study, in vitro experiments were conducted to compare the physical characteristics between collagen membranes with and without cross-linking. Additionally, clinical outcomes were compared to determine the effect of cross-linking on bone regeneration and the preservation of bone grafts.

The degradation test revealed relevant differences in degradation rates between 4 and 12 h. To evaluate early-phase degradation, a 12 h enzymatic exposure protocol was adopted [28,29]. The selected time point was considered sufficient to assess early membrane resistance, which plays a critical role in initial GBR healing. Compared with the non-cross-linked collagen membrane (group A), the cross-linked collagen membrane (group B) exhibited a slower degradation rate. This finding suggests that group B is expected to retain its function for a longer duration in clinical applications compared with group A.

Clinically, the significance of the degradation period of barrier membrane biomaterials lies in maintaining sufficient time to support the induction and new bone formation of the GBR technique. Brunel et al. and Mattson et al. reported that the rate of absorption of collagen membranes is determined by the degree of collagen cross-linking; therefore, membranes with greater cross-linking and prolonged absorption times are expected to enhance bone regeneration [30,31]. Furthermore, the relatively low resistance to degradation exhibited by non-cross-linked collagen membranes may render them insufficient for effectively supporting bone regeneration [32]. The clinical significance of cross-linked collagen membranes lies in their ability to maintain structural integrity during the bone formation period, thereby facilitating effective performance in GBR procedures.

However, other studies have reported contradictory results. Cross-linked collagen may negatively impact tissue regeneration and vascularization [33,34]. Similarly, these membranes exhibit higher postoperative exposure and complication rates, which could adversely affect bone regeneration [25]. Although our study did not observe such complications, these findings suggest that cross-linking-induced modifications may limit clinical applicability in certain cases.

The tear strength in group B was more than four times greater than that in group A. Although the number of tests performed was insufficient for statistical analysis, the findings suggest that the tear strength of cross-linked collagen membranes exceeded that of non-cross-linked collagen membranes. The structural characteristics of cross-links contribute to the enhanced mechanical stability of collagen membranes, as a higher cross-link density increases the number of interconnected collagen molecules, thereby reinforcing the matrix and improving its stiffness under deformation [35,36].

The tear strength test in this study was designed to approximate the clinical conditions by securing the collagen membrane to a bone block using a pin, creating a localized stress concentration near the fixation site. This setup aimed to reflect the mechanical challenges faced during GBR, where membranes are stabilized over bone using pins or surrounding biological tissue. Thus, the measured tearing resistance provides insight into both material durability and clinical handling performance. The study demonstrated that cross-linked collagen membranes exhibited greater tear resistance, which is considered an important factor for enhanced clinical application and usability, such as improved stability when using membrane fixation methods like pins or anchors. Membranes with high pull and tear resistance tend to show favorable handling properties during placement procedures [25]. Moreover, greater tensile strength in collagen membranes is associated with enhanced fixation and plasticity during surgical procedures, which may contribute to improved stabilization over large defects [37]. However, due to differences in physical characteristics compared to conventional non-cross-linked membranes, clinicians may not be readily accustomed to their handling. A short learning curve may therefore be necessary, which could present a limitation in early clinical adoption.

Considering both the introduction and the results of the cytotoxicity test, thorough removal of cross-linking agents is essential to ensure biocompatibility. In this study, the cross-linked collagen membrane was fabricated using the EDC/NHS method, which is widely recognized for its low cytotoxic potential and favorable biocompatibility in clinical applications. Based on the results of the in vitro cytotoxicity test, it appears that this requirement was adequately met, suggesting that the cross-linked collagen membrane used in this study exhibits acceptable biocompatibility.

Collectively, the results from the three in vitro experiments suggest that the cross-linked collagen membrane demonstrated biocompatibility comparable to that of the non-cross-linked membrane, with no evidence of cytotoxic effects observed. Furthermore, based on the degradation and tear strength tests, the cross-linked membrane exhibited superior physical properties, including greater tear resistance and slower degradation. These findings indicate that the cross-linked collagen membrane used in this study provides both comparable biocompatibility and enhanced physical performance relative to the conventional non-cross-linked membrane. Additionally, cross-linking methods have also been explored in bioadhesive systems, primarily to enhance the mechanical properties and stability. While these materials differ from collagen-based membranes in both composition and application, their use of cross-linking without significant cytotoxicity may suggest broader applicability of such approaches across biomaterials [38].

In this study, collagen membranes were used to cover cystic lesions. Chiapasco et al. reported that osteogenesis of the jawbone requires more than 12 months, with larger defects necessitating up to 24 months for complete osteogenesis [39]. Therefore, membranes used in GBR should maintain their structural integrity for at least 3–9 months, and those with a higher degree of cross-linking are capable of preserving the membrane structure over extended periods. Moreover, stable membranes are essential to prevent collapse of the grafted bone and surrounding soft tissue [40]. By contrast, premature membrane resorption of the membrane may lead to incomplete bone healing [41]. Previous studies have indicated that when absorbable membranes are used for GBR, they must remain functional for at least 6 months to support new bone formation [42,43]. The preservation of the bone wall and protection of the defect are important, and the use of cross-linked collagen membranes with enhanced resistance to degradation may offer significant advantages during this period. As described above, the ideal degradation timeline of collagen membranes in bone regeneration varies depending on the clinical context and specific application. Although a degradation period of at least 3 months is generally required, cases involving cystic lesions or large defects may necessitate a duration of 12 months or longer [40,42,43]. In this context, group B in the present study may serve as a favorable option for various clinical GBR applications, including large defects such as cystic lesions.

In the clinical study, CBCT data were used to compare outcomes between the two types of collagen membranes. CBCT was selected as the primary outcome metric due to its ability to provide accurate, three-dimensional, and non-invasive assessment of bone regeneration [44,45]. These references support the use of CBCT as a valid and practical tool for quantitative assessment of bone regeneration in clinical studies.

Additionally, minimal differences were observed in bone volume ratios between the membrane types, with no significant differences detected. These findings suggest that both cross-linked and non-cross-linked collagen membranes demonstrate comparable bone formation capabilities of GBR process. These findings are consistent with those reported in previous studies [25,46,47]. As a reference to similar studies, cross-linked collagen membranes may exhibit resistance to bacterial degradation, even when prematurely exposed to the oral environment [21].

From another perspective, although the cross-linked collagen membranes demonstrated superior physical characteristics, such as enhanced tear strength and slower degradation rates in vitro, these advantages did not translate into significantly improved clinical outcomes in this study. This discrepancy may be attributed to the fact that mechanical properties alone do not fully determine the biological performance of membranes in vivo. Several studies, including systematic reviews and randomized controlled trials, have reported no statistically significant differences in bone regeneration between cross-linked and non-cross-linked collagen membranes, suggesting that factors such as tissue integration, vascularization, and host response may play a more critical role than degradation resistance or handling strength in clinical success [25,48].

This study has some limitations. First, the products used in group A and B differed not only in cross-linking treatment but also in origin, making it difficult to attribute GBR outcomes solely to cross-linking. Specifically, bovine-derived and porcine-derived collagen membranes were used in both group A and B. These are widely used, and the biocompatibility of the cross-linked collagen membrane was demonstrated in the present study through in vitro cytotoxicity testing, which confirmed an appropriate level of cell viability. In addition, our in vitro degradation and tear resistance tests demonstrated that the cross-linked collagen membrane exhibited a slower degradation rate and greater tear resistance compared to the non-cross-linked membrane, indicating improved physical stability. Nonetheless, membrane properties can vary significantly based on the source species of collagen (e.g., bovine or porcine) and the processing techniques applied [49]. As such, ideally, membranes that differ solely in cross-linking status while sharing identical composition and processing parameters would be used to isolate the effect of cross-linking. However, obtaining such products is rarely feasible in clinical settings. Second, the in vitro degradation test was conducted with only one sample per membrane type. Although the method was expected to yield consistent trends regardless of repetition, the lack of replicates limits the statistical reliability of the findings. Future studies should include adequate sample sizes to enable quantitative validation. Last, a larger cohort and extended observation periods would provide more robust and generalizable results.

This study has some advantages. First, bone formation was evaluated and compared in three dimensions. Volumetric changes in the bone grafts and the extent of new bone formation were assessed using two different methods. Additionally, the efficacy of cross-linked collagen membranes for bone grafting in defects was evaluated without distinguishing between the maxilla and mandible. In patients with maxillary periapical cysts, in whom the risk of graft or membrane exposure is low and bone regeneration capacity is generally high, minimal differences between the groups may be expected. In those with mandibular defects, particularly in the molar region, the risk of exposure is high, making membrane functionality and safety more critical. By randomly including both maxillary and mandibular defects, this study offers broadly applicable insights.

In future research, several directions may be considered. This study primarily focused on short-term clinical outcomes within a 6-month follow-up period; thus, long-term studies are warranted to investigate potential delayed complications and the sustained efficacy of cross-linked membranes. Moreover, having confirmed the clinical safety and biocompatibility of the tested cross-linked membrane, it would be meaningful to explore evaluation models that consider both clinical performance and cost-effectiveness.

Despite differing views on cross-linking, the use of cross-linked and non-cross-linked collagen membranes in the treatment of common cystic defects has proven to be clinically effective and stable. Although cross-linking enhances the biodegradation resistance and tear strength of the collagen membrane, the bone regeneration outcomes are comparable to those achieved with non-cross-linked collagen membranes.

## 5. Conclusions

The cross-linked collagen membrane exhibits superior physical properties compared to the non-cross-linked membrane and demonstrates non-inferior biocompatibility. Therefore, both membranes can be appropriately selected and applied depending on the clinical situation and their respective advantages of bone graft for GBR. Further long-term studies and large-scale clinical evaluations are needed to more thoroughly assess the efficacy and safety of cross-linked collagen membranes.

## Figures and Tables

**Table 1:** Schedule of clinical evaluations and imaging procedures during the study period.

Assessment	Screening	Surgery	2 W Follow-Up	3 M Follow-Up	6 M Follow-Up
Panoramic radiograph	O	O	O	O	O
Cone-beam CT	O		O		O
Clinical photographs		O	O	O	O
Adverse event monitoring	O	O	O	O	O

W = weeks; M = months.

**Table 2:** Degradation rate with remaining weight percentage and tear strength (N) of non-cross-linked (group A) and cross-linked (group B) collagen membranes.

		Group A	Group B
		Degradation rate (%)	
Time (hours)	0	0.0	0.0
	2	57.9	21.5
	4	100.0	34.1
	6	100.0	45.7
	12	100.0	79.5
		Tear strength (N)	
*n* = 3	1	4.2	18.4
	2	4.5	19.4
	3	3.3	17.3
Average ± SD		4.0 ± 0.6	18.4 ± 1.1

Degeneration rate after exposure to collagenase solution (50 U/mL) over time. Group B showed significantly lower degradation state and higher tear resistance than group A, with a mean tear resistance approximately 4.6 times higher.

**Table 3:** Cytotoxicity evaluation based on absorbance measurement.

Sample	Result: *n* = 6						Average
Negative	3.724	3.757	3.809	3.633	3.276	3.290	3.58 ± 0.12
Positive	0.554	0.597	0.536	0.440	0.465	0.474	0.51 ± 0.09
Group B	3.051	3.098	3.094	3.120	2.889	2.937	3.03 ± 0.05

Group B showed approximately 84.6% cell viability relative to the negative control, exceeding the 70% threshold for non-cytotoxicity.

## Data Availability

The original contributions presented in this study are included in the article/Supplementary Materials. Further inquiries can be directed to the corresponding author(s).

## Supplementary Materials

The following supporting information can be downloaded at: https://www.mdpi.com/article/10.3390/bioengineering12080876/s1, Figure S1: Volumetric analysis of bone regeneration using cone-beam computed tomography (CBCT) images. (**A**) Postoperative CBCT view; (**B**) CBCT view at 6-month follow-up; (**C**) delineation of defect volume on postoperative CBCT; (**D**) delineation of defect volume on 6-month follow-up CBCT; (**E**) volume of high-density area on postoperative CBCT; (**F**) volume of high-density area on 6-month follow-up CBCT; (**G**) both the high-density area and remaining defect volume at 6-month follow-up; (**H**) volume of reduced defect observed at 6-month follow-up.

## Abbreviations

The following abbreviations are used in this manuscript:

GBR	Guided bone regeneration
CBCT	Cone-beam computed tomography
