# Supplementary material for: Efficacy of Cross-Linked Collagen Membranes for Bone Regeneration: In Vitro and Clinical Studies
Source: Bioengineering (Basel). 2025 Aug 14;12(8):876. doi: 10.3390/bioengineering12080876 (PMC12383682; doi:10.3390/bioengineering12080876)
Supplement: Supplementary file 1 [file bioengineering-12-00876-s001.zip › Figure_S1___Legend.pdf]

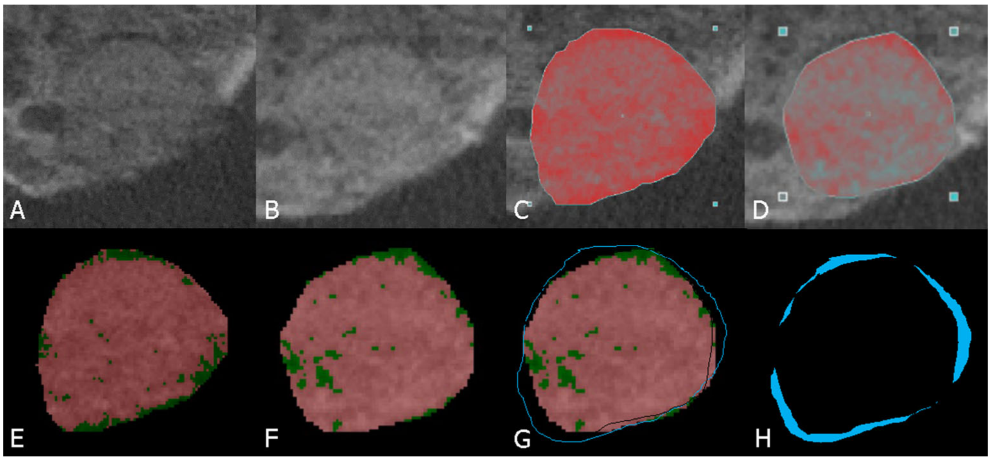

Figure S1. Volumetric analysis of bone regeneration using cone-beam computed tomography (CBCT) images. (A) Postoperative CBCT view; (B) CBCT view at 6-month follow-up; (C) delineation of defect volume on postoperative CBCT; (D) delineation of defect volume on 6-month follow-up CBCT; (E) volume of high-density area on postoperative CBCT; (F) volume of high-density area on 6-month follow-up CBCT; (G) both the high-density area and remaining defect volume at 6-month follow-up; (H) volume of reduced defect observed at 6-month follow-up.
